# The Mickey Mouse problem: Distinguishing religious and fictional counterintuitive agents

**DOI:** 10.1371/journal.pone.0220886

**Published:** 2019-08-08

**Authors:** Thomas Swan, Jamin Halberstadt

**Affiliations:** Department of Psychology, University of Otago, Dunedin, New Zealand; Middlesex University, UNITED KINGDOM

## Abstract

The Mickey Mouse problem refers to the difficulty in predicting which supernatural agents are capable of eliciting belief and religious devotion. We approached the problem directly by asking participants to invent a “religious” or a “fictional” agent with five supernatural abilities. Compared to fictional agents, religious agents were ascribed a higher proportion of abilities that violated folk psychology or that were ambiguous–violating nonspecific or multiple domains of folk knowledge–and fewer abilities that violated folk physics and biology. Similarly, participants rated folk psychology violations provided by the experimenter as more characteristic of religious agents than were violations of folk physics or folk biology, while fictional agents showed no clear pattern. Religious agents were also judged as more potentially beneficial, and more ambivalent (i.e., similar ratings of benefit and harm), than fictional agents, regardless of whether the agents were invented or well-known to participants. Together, the results support a motivational account of religious belief formation that is facilitated by these biases.

## Introduction

The domain of counterintuitive agents includes all manner of gods, goddesses, superheroes, and cartoon characters with abilities and features that violate our intuitive or ‘folk’ expectations about the world [1]. An omnipresent god would, for example, violate the ‘folk physics’ expectation that objects cannot be in several places at once. An immortal superhero violates the ‘folk biology’ expectation that all living things die. A talking mouse violates the ‘folk psychology’ expectation that animals cannot use language. These intuitive expectations about the features and behaviors of stimuli from various ontological categories (i.e., objects, living things, animals) are described as maturationally natural because they appear early in development and across cultural contexts [2–4]. We implicitly apply them to our environment, such that any counterintuitive agent that violates them is likely to attract attention.

Prior research has shown that agents violating a minimal number of intuitive expectations (“minimally counterintuitive,” or MCI, agents) are typically remembered better than agents that adhere to expectations, dramatically violate intuitive expectations, or only violate culturally acquired expectations [1, 5–10]. As it could be argued that gods and goddesses are MCI agents, some authors have implicated this memory bias in the development and pervasiveness of religion [11–13]. Indeed, counterintuitive statements are rated more ‘religious’ than intuitive statements [14] and a minimal amount of such content may be an ideal condition for belief [15–17]. Others, however, have asked why fictional agents such as Mickey Mouse and Santa Claus aren’t believed and worshiped to the same extent as gods, even though they are comparably counterintuitive [18]. The “Mickey Mouse problem” thus came to describe the difficulty of current cognitive theories of religion to predict which MCI agents are more likely to be believed, and in turn become candidates for religious devotion.

Responses to the Mickey Mouse problem have been limited in number and scope. Some authors suggest there *is* no way to distinguish the content of religious and secular MCI agents, attributing religious belief instead to contextual conditions, such as conformity with cultural norms, mimicry of prestigious and authoritative individuals, and observation of costly displays of commitment that enhance the credibility of the performer’s beliefs [19–24]. This approach is unsatisfying, however, both because religious and nonreligious agents have not been systematically compared on dimensions other than their counterintuitiveness, and because distinctions in terms of “context” only push back explanation one step. While context may be important for explaining which agent from a set of established religious agents is worshipped at a particular time and place (known as the “Zeus Problem” [20]), it does not explain how contexts become established or favorable toward particular agents in the first place [25–27].

Solving the Mickey Mouse problem may therefore require a more thorough examination of the features of believable/worshipped and secular/fictional counterintuitive agents. Previous work has alluded to differences that might be important in this regard. For example, people tend to ascribe the Christian god analogical and abstract features (e.g., “God is love”; “God is the beginning and end”) that potentially violate multiple domains of folk expectations, but ascribe non-divine entities more concrete abilities that violate specific folk domains (e.g., “reads minds” only violates folk psychology; “teleports” only violates folk physics) [17]. Indeed, an “all-seeing” god might achieve this feat through biological mutation, physical omnipresence, or psychological telepathy; a god that “is love” or “the beginning and end” might imply a physical transformation, or perhaps an absence of other intuitive mental or biological processes. We call these nonspecific features and abilities that, nevertheless, appear to require a degree of counterintuitiveness, “ambiguous violations”. If common to gods, their role in religious belief may be to afford gods influence over a wider range of events than is possible for non-divine agents, and to allow individuals greater scope for interpretation and motivated reasoning about the causes and purposes of those events, including apparently disconfirming events (e.g., via appeals to God’s “mysterious ways”) [28, 29].

Harmon-Vukic [17] also uncovered a second feature of religious agents: an interest in human affairs. Participants tended to assign God abilities, roles, and relationships that indicated a direct interest in people’s lives, such as inspiring emotions and playing a beneficent role as teacher, friend, or healer. Similarly, Pyysiäinen et al. [14] found that counterintuitive statements were rated more “religious” than intuitive statements, especially when those statements involved agents interested in human affairs. Such an interest might suggest the importance of religious agents’ psychological abilities; agents that, for example, exist as disembodied minds [30], who have the ability to impose their will on individuals’ behavior [31], or are omniscient [32], may be better placed to influence human affairs than those who violate only physical (e.g., omnipresence) or biological (e.g., immortality) intuitions. Indeed, a number of studies have documented religious agents’ violations of folk psychological beliefs in particular (e.g., [33, 34]).

Religious agents’ interest in human affairs may be seen in light of a more general principle: their relevance to human fitness. Religious agents may be encoded and, ultimately, worshipped, because they serve intelligible purposes [24], and perhaps represent a potential threat or benefit to human survival and/or reproductive prospects. Indeed, in prior research, we found that counterintuitive agents both pose a greater threat, and also offer greater opportunity (i.e., hold greater potential benefits) than intuitive agents, and it may be that fitness relevance further distinguishes religious and fictional agents [35].

In particular, the idea that potential benefits afforded by gods make them attractive targets for belief is reminiscent of the popular claim that emotional or motivational processes are key ingredients in religious belief (e.g., [36]). Though largely ignored or criticized in cognitive theories of religion [11], the claim is supported by several studies in which negative affective states seemingly motivate people to seek out comforting religious beliefs about beneficent, overseeing gods [37–45]. If this motivational hypothesis–that people consciously or unconsciously *want* to believe in gods because they fulfil some psychobiological need–is correct, religious agents should be perceived as more of a potential benefit than fictional agents.

Operationalizing fitness relevance in terms of an entity’s potential threat and potential benefit to human well-being, as opposed to a list of apparently fitness relevant criteria (e.g., “possesses socially strategic information” and “interacts with the human world” [46, 47]), permits further predictions. For example, monotheistic gods are commonly described as ambivalent, exacting both love and fear (e.g., "What doth the Lord thy God require of thee, but to fear the Lord thy God, to walk in all his ways, and to love him”; Deuteronomy 10:12)), which might partially explain their cultural evolutionary success over polytheistic deities [48], as well as over fictional agents not worshipped by anyone, who appear to be more valenced. Thus, it may be that the most believable counterintuitive agents are those that are capable of both threatening and rewarding adherents.

In sum, there are both theoretical and empirical reasons to expect that the content of religious versus secular counterintuitive agents will differ systematically in ways that illuminate the process of religious belief formation. Specifically, we hypothesize that participants will more likely ascribe psychological violations and ambiguous violations to religious than to nonreligious agents. We also predict that religious agents will be judged more potentially harmful, more potentially beneficial, and more ambivalent (i.e., similar ratings of harm and benefit), compared to nonreligious agents. In the following study, we test these hypotheses and construct a template that describes the pantheon of religious counterintuitive agents.

## Method

### Participants

Consistent with an a-priori power analysis of previous studies that measured ratings and recall for counterintuitive abilities [14, 35], we aimed to recruit at least 276 participants from Amazon’s Mechanical Turk, an online community of “workers” who perform simple tasks online in exchange for token payment (US$.80 in this case). Three hundred and nine participants were ultimately recruited (139 female, 168 male, and 2 identifying as “other”). All were native English-speaking U.S. nationals currently living in the U.S. Their average age was 33.6 years (SD = 9.7); 160 identified with a religion (of which 89% were Christian); the remaining identified as “agnostic”, “atheist”, or “none”.

### Materials

Stimuli included twenty minimally counterintuitive abilities that described a single breach of folk physics, folk biology, or folk psychology (see Table 1), based on content domains defined previously [1, 49]. In prior research, participants answering the question “Do you think a human being could possess this ability?” rated these abilities significantly less intuitive (M = 1.34, SD = .58) than a set of twenty normal (intuitive) abilities (M = 5.84, SD = 1.52; r = .95, p < .001) [35: Study 1].

**Table 1:** Counterintuitive abilities used in this study.

Ability	Violation type
Disintegrate any object they touch.	Physics
Reverse the direction of time.	Physics
Split into an army of duplicate beings.	Physics
Walk right through walls and people.	Physics
Levitate and fly unaided through the air.	Physics
Evaporate when they feel heavy.	Physics
Be in two places at the same time.	Physics
Raise the dead and command them.	Biology
Quickly grow to many times their size.	Biology
Transform themselves into a monster.	Biology
Create living, breathing people out of sand.	Biology
Remove their head and reattach it.	Biology
Never die and will live forever.	Biology
Give birth to different species of animal.	Biology
Send thunderstorms to villages that offend them.	Psychology
Directly control other people's minds.	Psychology
Hurl objects just by using their mind.	Psychology
Answer any question because they know everything.	Psychology
Converse with billions of people simultaneously.	Psychology
See the future and know what will happen.	Psychology

### Design and procedure

This research received ethics approval from the University of Otago’s Department of Psychology and the Human Ethics Committee (B–D16/055). The study conformed to the guidelines and principles of the Declaration of Helsinki and Amazon’s Participation Agreement. No further permits or approvals were required.

The study was administered in the Qualtrics survey environment and completed online. Participants completed one of two surveys, described to participants as investigating “the features of religious [fictional] beings and entities”. Unless otherwise noted, the surveys differed only in whether the word “religious” or “fictional” was used in the instructions and dependent measures.

After providing written informed consent, participants completed three tasks, always in the same order. First, they were asked to “invent a new religious [fictional] being or entity with supernatural abilities,” and to list five supernatural abilities they thought it would possess. Afterwards, they were re-presented with the abilities they generated and asked to rate each one in terms of its counterintuitiveness (“do you think a human being could possess this ability?”). Participants were then asked to imagine a real encounter with the entity they invented, and to rate the potential threat (“do you think it could cause you significant harm if it wanted to?”) and benefit (“do you think it could significantly improve your life if it wanted to?”) it posed. All ratings in this study were made on scales anchored at 1 (not at all) and 7 (definitely).

Second, participants were presented with the abilities in Table 1, in a single, randomly-ordered “list of supernatural abilities that might apply to religious [fictional] beings and entities”. For each ability, participants provided an attribution rating by answering “is this ability likely to be attributed to a religious [fictional] being or entity?”

Third, participants were asked to “name 5 well-known religious [fictional] beings or entities with supernatural abilities, from the past or present.” We defined a religious agent as an entity “that many people believe exists, and that is part of a religion”, and a fictional agent as an entity “that people generally do not believe exists”. Participants then rated each entity in terms of its potential harm and benefits, answering the questions specified above. They were told to assume, for the purposes of making their ratings, that their five agents do exist.

Prior to being debriefed, participants were administered an attention check, requiring that they recall the type of agent (religious or fictional) they invented earlier in the survey. Fifteen participants failed to answer this question correctly and were not included in any of the following analyses of the data set [50].

## Results

### Participant-invented agents

Eight participants were excluded from analyses involving the five abilities they chose for their new entity because none of their abilities could be coded as counterintuitive. In all, 141 participants chose 702 abilities for religious agents (two participants provided fewer than five abilities, but were retained in the analysis), and 145 participants chose 725 abilities for fictional agents. The abilities participants chose were coded by the first author and a hypothesis-blind research assistant in terms of whether they (1) exclusively violated folk psychology, (2) exclusively violated folk biology, (3) exclusively violated folk physics, (4) violated nonspecific or multiple folk domains (i.e., ambiguous), or (5) violated none of the domains (i.e., was not a counterintuitive ability). Coders agreed 92% of the time, with disagreements resolved through discussion.

As abilities more strongly associated with religious [fictional] agents are likely to be generated first, each ability was weighted by (6-k)/5, where *k* is its position within the participant’s list of five abilities. This transformation assigns a “salience score” (i.e., 1.0, 0.8, 0.6, 0.4, and 0.2 to items 1 to 5 respectively) [51] that effectively captures the ease with which a violation type comes to mind when thinking of a religious [fictional] agent. For example, if the participant first listed three abilities that were folk biology violations followed by two that were folk physics violations, salience scores would be 2.4 and 0.6 for these violation types respectively, and zero for the three unlisted types. This procedure, which is a standard technique for dealing with free-list data in the anthropological study of religion (e.g., [52]), has the additional advantage of controlling for the number of abilities listed. As the average number of abilities provided by participants differed between religious and fictional agents, salience scores are reported as percentages of total salience scores for the violation type categories included in a given analysis.

As expected (since their task was to list supernatural abilities), participants listed relatively few intuitive abilities, and their mean salience did not differ between religious (M = .13; SD = .21) and fictional agents (M = .10; SD = .17; p = .231). Participants also listed relatively few ambiguous violations, but such abilities were far more salient for religious (M = .16; SD = .20) than for fictional agents (M = .01; SD = .06), t(165.26) = -7.85, p < .001, r = .52.

**Fig 1:**
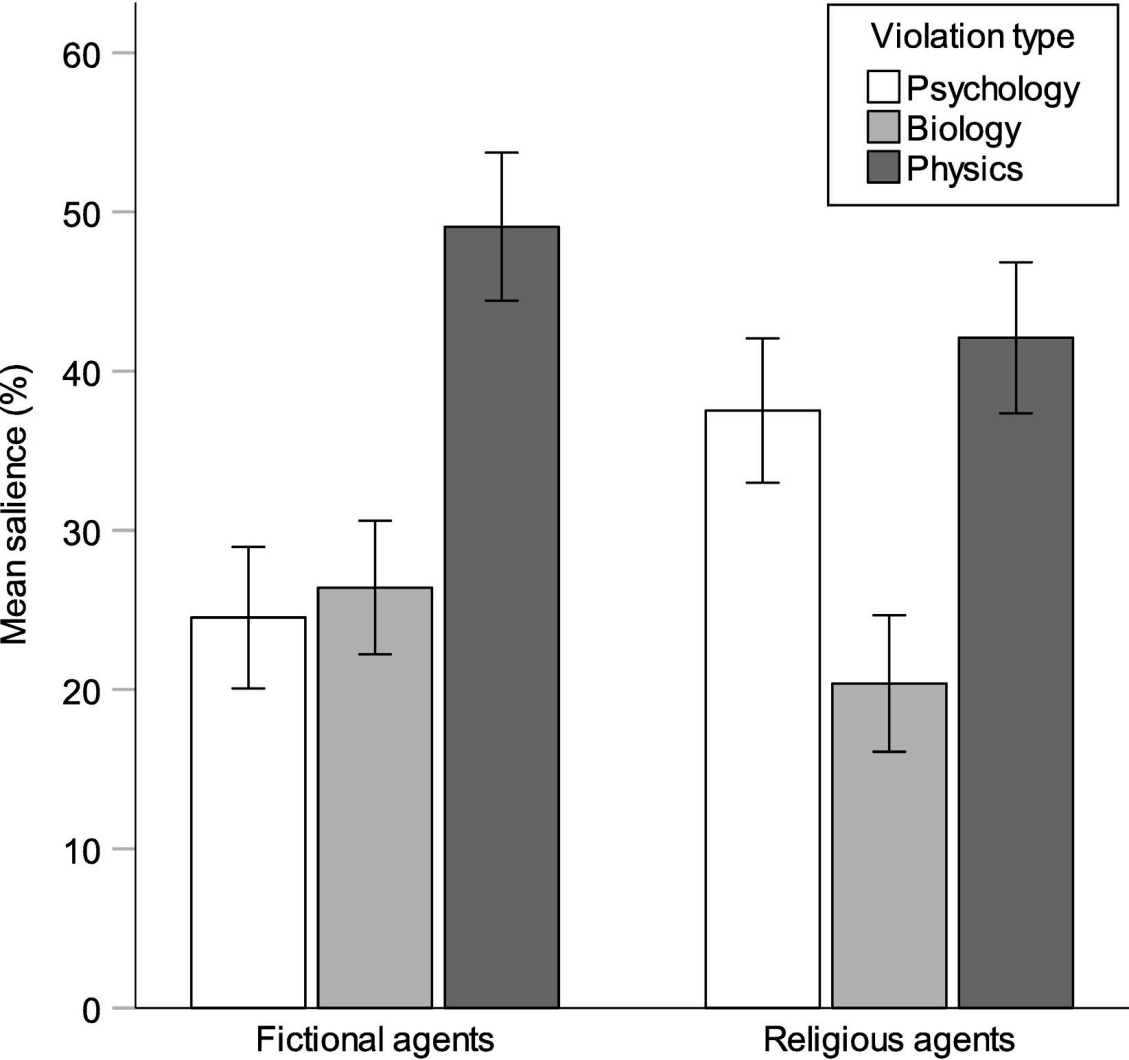
Mean salience of folk psychology, folk biology, and folk physics violations for fictional and religious agents, treated as percentages of total salience.

Salience scores for specific domain violations were submitted to a 2 (agent type) x 3 (violation type) mixed model ANOVA, with the second factor treated as a repeated measure; results appear in Fig 1. There was a main effect of violation type, F(2, 564) = 32.72, p < .001. Bonferroni-corrected pairwise comparisons showed that physics violations (M = .46; SD = .29) were more salient than psychology violations (M = .31; SD = .28; p < .001) or biology violations (M = .24; SD = .26; p < .001); psychology violations also differed from biology violations (p = .013). Although not a focus of the current study, this ordering replicates other studies that have found better recall for folk physics violations over other violation types [9, 35]. Of more interest, there was an interaction between agent type and violation type, F(2,564) = 8.17, p < .001. Looking at each violation type independently, t-tests indicated that psychology violations were more salient for religious agents than for fictional agents, t(282) = -4.03, p < .001 r = .23, while biology and physics violations were more salient for fictional agents, t(282) = 1.98, p = .049, r = .12, and t(282) = 2.07, p = .039, r = .12. These latter two effects were not significant at a Bonferroni-adjusted alpha level (although this adjustment may be unnecessary [53]).

**Fig 2:**
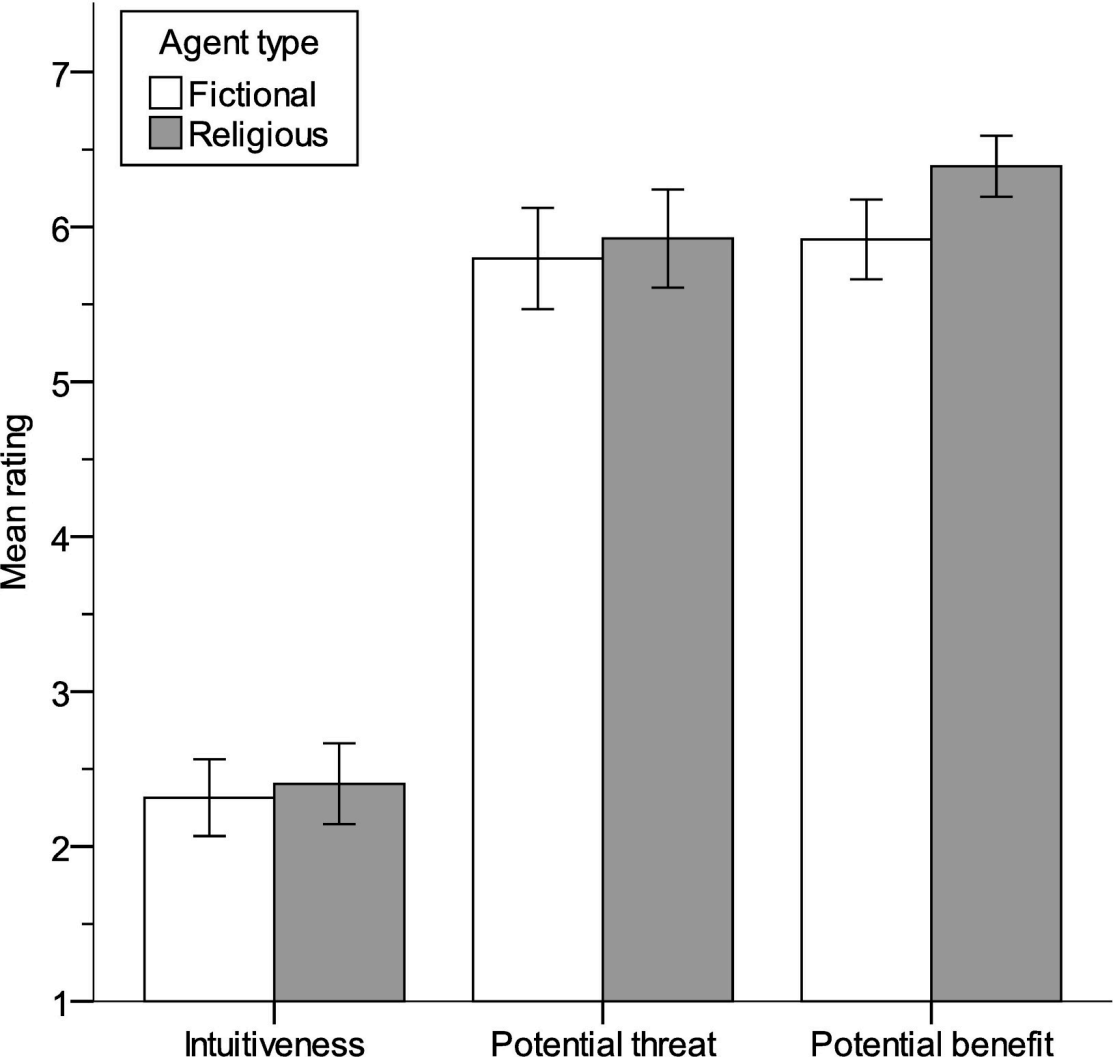
Intuitiveness, threat, and benefit ratings for fictional and religious agents.

Thirteen participants were excluded from analyses involving their intuitiveness, threat, and benefit ratings for the agent they invented after giving insufficient attention to the questions (i.e., responding faster than 1s per item, a criterion set a priori). Mean intuitiveness, threat, and benefit ratings for participant-invented agents are shown in Fig 2. Fictional and religious agents did not differ significantly on intuitiveness (p = .583), or potential threat (p = .602), but potential benefit ratings were higher for religious agents, t(255.14) = -2.84, p = .005, r = .18.

We defined the “ambivalence” of each agent in terms of the absolute difference between its threat and benefit rating, such that larger values reflect relatively polarized ratings of threat and benefit. On average, religious agents (M = 1.02; SD = 1.77) were rated more ambivalent than fictional agents (M = 1.56; SD = 1.99), t(268.08) = 2.35, p = .020, r = .14. Thus, for fictional agents, mean threat and mean benefit ratings were only comparable (see Fig 2) because participants’ beneficent heroes and threatening villains cancelled each other out.

### Ability attribution ratings

**Fig 3:**
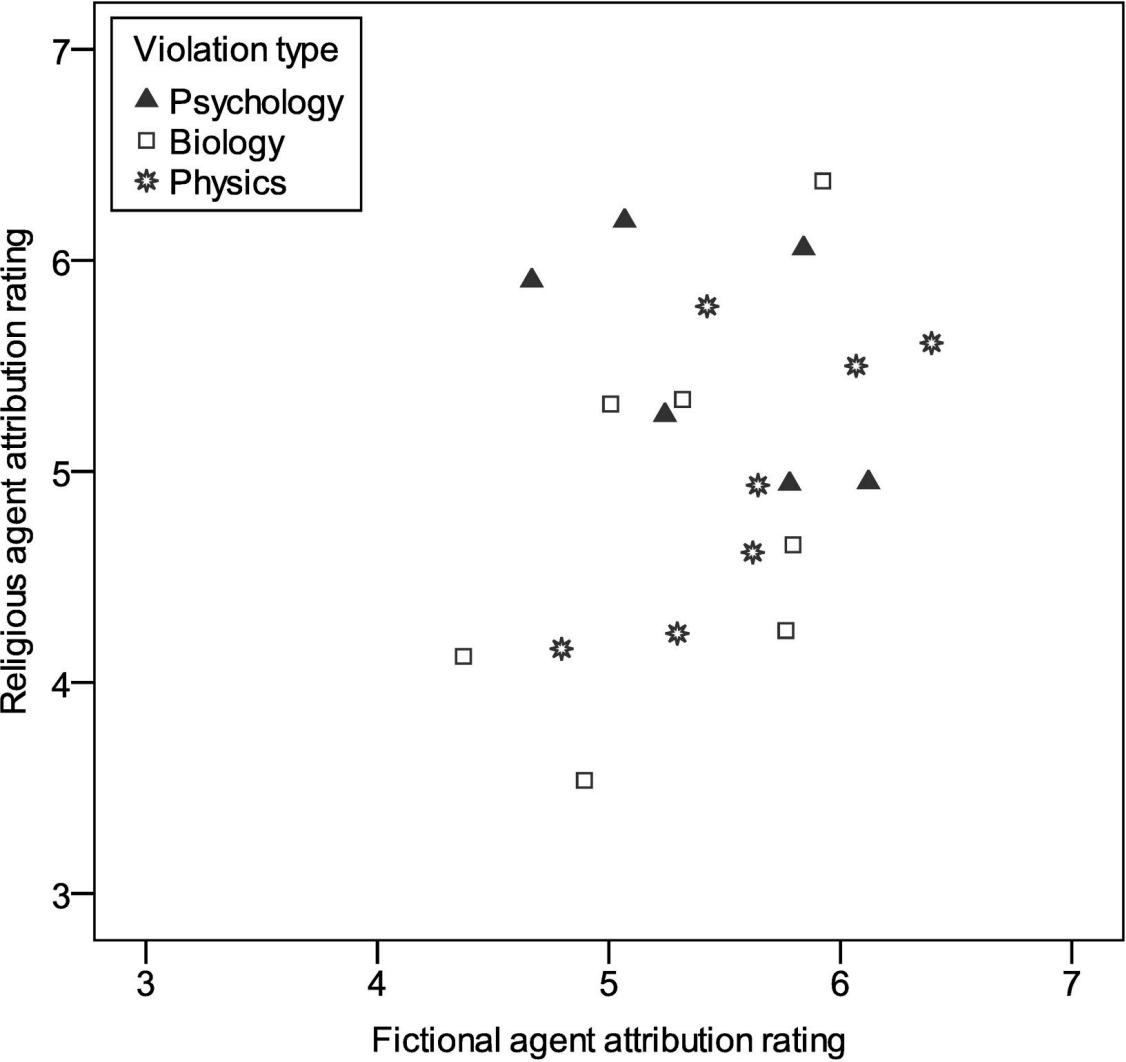
Attribution ratings for 20 counterintuitive abilities (see Table 1) to religious and fictional agents. Abilities towards the upper-left portion of the graph are more attributable to religious agents.

Attribution ratings (i.e., whether the supernatural abilities in Table 1 apply to religious and fictional beings) were submitted to a 2 (agent type) x 3 (violation type) mixed model ANOVA, with the second factor treated as a repeated measure. (Twenty-four participants were excluded from this analysis for inattention to the task.) There were theoretically uninteresting main effects of agent type, F(1,268) = 3.99, p = .047, and violation type, F(2,536) = 34.58, p < .001, with Bonferroni-corrected pairwise comparisons indicating that the abilities were more strongly associated overall with fictional agents (M = 5.45; SD = 1.40) than with religious agents (M = 5.09; SD = 1.44; p = .047), and that psychology violations (M = 5.50; SD = 1.38) were more strongly associated with agents generally than physics violations (M = 5.28; SD = 1.54; p < .001) and biology violations (M = 5.04; SD = 1.62, p < .001), which also differed from one another (p < .001). Of more interest, there was an interaction between agent type and violation type, F(2,536) = 25.14, p < .001 (see Fig 3). For religious agents, Bonferroni-corrected pairwise comparisons indicated that psychology violations (M = 5.55; SD = 1.44) were more strongly associated than biology (M = 4.80; SD = 1.57; p < .001) or physics violations (M = 4.98; SD = 1.56; p < .001), which did not significantly differ (p = .056), but displayed a trend consistent with the main effect of violation type. For fictional agents, psychology violations (M = 5.45; SD = 1.30) did not differ significantly from biology (M = 5.30; SD = 1.65; p = .245) or physics violations (M = 5.61; SD = 1.46; p = .160), while physics violations were more strongly associated than biology violations (p < .001).

### Well-known agents

Fourteen participants were excluded from analyses involving their 5 well-known agents because they didn’t specify any agents that matched the requested criteria; twenty-three were excluded due to inattention to the rating questions. Of the remaining participants, 128 provided a total of 518 fictional agents (108 distinct agents), and 129 provided a total of 591 religious agents (64 distinct agents). A list of these agents and their mean ratings can be found in the supporting information (S1 Table). These mean ratings (across agents) suggest the prevalence of “God” and “Jesus” in participants’ responses did not significantly affect the results (across participants) detailed below.

**Fig 4:**
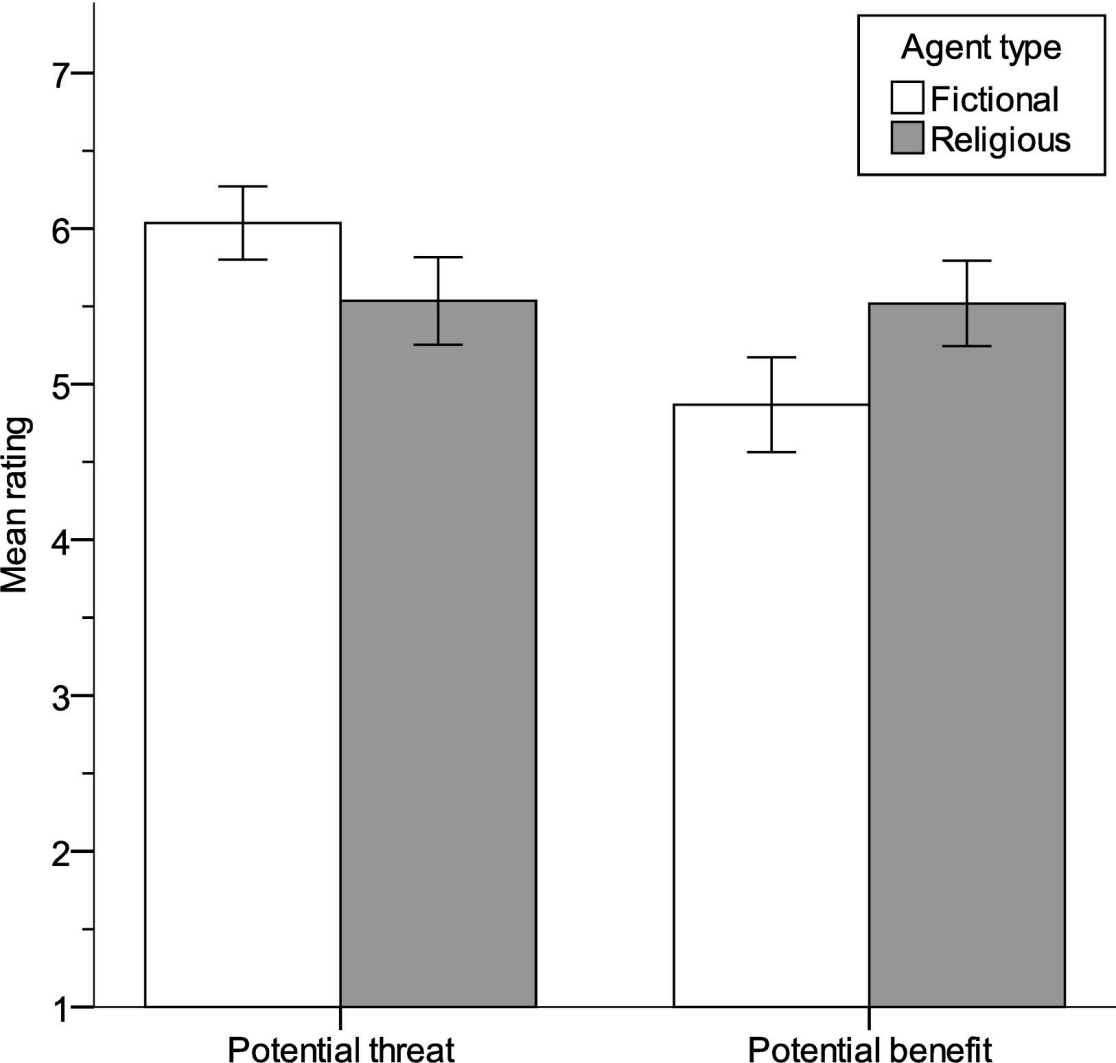
Mean threat and benefit for well-known fictional and religious agents.

As seen in Fig 4, religious agents were rated less threatening, t(255) = 2.69, p = .008, r = .17, and more of a benefit, t(255) = -3.12, p = .002, r = .19, than fictional agents. Ambivalence was calculated as described above; religious agents (M = 1.30; SD = 1.38) were again rated more ambivalent than fictional agents (M = 2.07; SD = 1.74), t(255) = 3.90, p < .001, r = .24.

## Discussion

Cognitive scientists of religion still don’t know why only some counterintuitive agents are believable and worthy of devotion. We examined the characteristics of religious and nonreligious supernatural agents directly and found four ways in which they differ. First, counterintuitive abilities that violate folk psychology were more salient for participant-invented religious agents than for nonreligious agents and were more closely associated with religious agents than other types of violations. Second, counterintuitive abilities that were ambiguous–violating nonspecific or multiple folk domains–were more salient for invented religious agents than for nonreligious agents. Third, both invented and well-known religious agents were rated more of a potential benefit than their nonreligious counterparts. Fourth, both invented and well-known religious agents were more ambivalent (i.e., the difference between a religious agent’s threat and benefit rating was relatively small) than nonreligious agents, which were more valenced towards extremes of heroism (high benefit, low threat) or villainy (low benefit, high threat).

We suggest that these biases collectively provide a template for predicting which counterintuitive agents can become objects of religious devotion. In particular, the biases for more beneficial and less threatening abilities (the latter only for well-known agents) suggest that “successful” agents must also be motivationally compelling: minimally counterintuitive traits make agents memorable, but memorable agents must satisfy psychobiological needs to become gods. Such gods are not necessarily more “powerful” or “fitness relevant” overall; it is the beneficial traits that distinguish religious agents, while threatening traits may often be higher for nonreligious agents.

Religious agents’ tendency to violate folk psychology over other folk beliefs is also consistent with this motivational interpretation. In a previous study, we found that agents with abilities that violate folk psychology were rated more of a potential opportunity (i.e., more beneficial) than agents violating folk physics or biology [35]. We surmised that agents violating folk psychology represent an opportunity to gain access to socially strategic knowledge—an opportunity that is particularly salient given our recent evolutionary history in which social threats have become increasingly costly and ubiquitous [54]. Indeed, the fact that agents have greater scope to violate folk psychology expectations may account for why agents feature prolifically in religious narratives [55], and appear more than other ontological categories (e.g., objects) in statements rated religious [14].

Nevertheless, these data only provide partial support for the motivational hypothesis. A motivation to believe in agents with beneficial abilities would not necessarily culminate in the formation of religious beliefs via motivated reasoning (i.e., biased strategies for accessing, constructing, and evaluating beliefs [28]). Belief formation also likely depends on an individual’s personality, cultural environment, and/or developmental history, among other factors. For example, people with a strong or temporarily elevated “fear of death” might possess a stronger motivation to believe in immortal beings than people with less fear [37, 38]. We consider it likely that at least some people form religious beliefs via a motivational path [37–45], however, widespread belief would depend on the commonality and thresholds of these individual and cultural factors.

The ambiguous traits used to describe religious agents, while not necessarily motivationally compelling in and of themselves, may interact with other motivational states to facilitate belief. When traits are defined ambiguously, abstractly [56], or metaphorically [57, 58] it becomes easier to attribute them in motivationally attractive ways. For example, the “better than average effect,” in which people rate themselves above average on positive traits and below average on negative traits [28, 59], is smaller when traits are precisely defined (e.g., neat, athletic, sarcastic, clumsy) than when they are ambiguous (e.g., idealistic, sophisticated, impractical, insecure) [29, 60], suggesting that ambiguity allows people to reason their way toward favored conclusions more easily. Similarly, the ambiguous content attributed to religious agents may facilitate motivated reasoning, making it easier to reason toward a belief in these agents. Counterintuitive abilities that can be demonstrated in a variety of ways (violating nonspecific or multiple folk domains), might permit gods to influence manifold situations of motivational significance [24], without precluding or disconfirming their involvement in any [26]. A god’s omnipotence, control of nature, magical powers, or “mysterious ways”, for example, can be applied in whatever manner a believer deems necessary.

A common argument against motivation-based theories of religion is that some gods are not comforting–they’re scary–and why would anyone want to believe in a scary god? We suggest that, like ambiguity, ambivalence facilitates the motivated reasoning process. We think anxiety and uncertainty attributed to threat-capable gods [61] can motivate belief-reinforcing behaviors, such as rituals and other deferential practices [45, 46, 62, 63], and that these behaviors become more intuitively compelling if the agent to whom they are directed is ambivalent. For example, rituals and prayers often depict a transaction in which a god is requested to perform a counterintuitive act in return for worship, good behavior, or a tangible offering. An entirely malevolent god would have little interest in accepting requests, just as an entirely benevolent god would have little interest in refusing them. Thus, an ambivalent god is the only god for whom transactional prayers and rituals make sense. Furthermore, an ambivalent god with whom we can communicate and occasionally extract positive outcomes may be more appealing, and more plausible, than a valenced god, or a god that acts capriciously or randomly. Thus, ambivalence should help make rituals and prayers an intuitively compelling avenue through which gods can deliver benefits (see [64] for other intuitively compelling ritual content), facilitating motivated reasoning towards a belief in these gods.

One anomalous finding is that well-known fictional agents were rated more threatening than well-known religious agents, but this wasn’t the case for invented agents. Negative information, such as about threatening agents, is more cognitively attractive than positive information [65]. A cultural unfolding of this negativity bias, constrained by the motivation to disbelieve overly threatening agents, may explain why it was restricted to culturally popular fictional agents. Such agents might share or even surpass the cognitive attractiveness of religious agents, in being counterintuitive *and* threatening, but may lack motivational attractiveness, resulting in popular but unbelievable beings. In other cases, threat may be lacking while benefit is not, which might result in motivationally attractive beings that don’t demand our attention for long enough to become religiously established (e.g., Santa Claus or the Easter Bunny). The cognitive attractiveness of threat may therefore be an alternative *or* additional reason why ambivalence appears to be a key feature of religious beings.

Nevertheless, it is adaptive to pay attention to threats, and possibly also to believe the reality of those threats in some contexts. In evolutionary terms, the cost of falsely believing a threatening agent is absent should be greater than falsely believing it is present, and this negative credulity bias [66] may explain why (an apparent minority of) religious agents deviate from ambivalence into malevolence [67]. Some cultural environments may require and activate our agency detection device more than others [68], making malevolent deities more likely.

Although American participants, mostly Christian, provided our data, we would expect similar results in other cultural contexts to the extent that they afford and foster similar motivations that are natural to the human mind (e.g., to avoid negative affective states). Conversely, cultural differences in the characteristics of religious agents may reflect motivations that arise or are more salient as a consequence of particular environments. Similarly, individual differences within cultures may reflect idiosyncratic motivations (e.g., for more social contact) associated with particular traits (e.g., death anxiety). This essentially motivational account of the characteristics of religious agents has the advantage of catering to the existence of atheists: despite observing the same content and contexts as everyone else, they lack belief, presumably because they lack a motivation for which this content is relevant.

Although the current study contributes to the development of a “belief template” that could, eventually, predict which agents are most likely to inspire religious devotion, it is just a start, with several acknowledged limitations. For example, we did not measure levels of belief in the well-known agents cited by participants, which could provide a better understanding of the role of individual differences. In addition, our distinction between religious and fictional entities neglects some counterintuitive agents, such as ghosts and spirits, that many people believe exist, but that are not necessarily objects of religious devotion. Future work might present participants with specific beings, entities, and other paranormal phenomena with a diverse range of a-priori plausibility before measuring belief and their perceived features. Similarly, our request for supernatural abilities restricted the number of *intuitive* features that participants attributed to their invented agents. Although we found no difference in rated intuitiveness or the number of intuitive abilities listed, the trend was towards religious agents being more intuitive, as found in other work [16, 17]. Finally, our proposed template neglects some religious agents, such as Satan and Loki, that are apparently neither beneficent nor ambivalent. It could be argued that belief in such agents (and polytheism in general) is declining, in line with the template’s proposed optimum, however, in less optimal settings, the ambivalence criterion might also be applied to groupings or entire pantheons of agents. Thus, we acknowledge that cultural and environmental factors may affect the reliability of the template, which is more a measure of cultural evolutionary success than a strict set of exclusionary criteria.

These limitations notwithstanding, we suggest a religious agent template, tentatively comprising beneficent yet ambivalent agents with ambiguous and folk-psychology-violating abilities, goes some way to solving the Mickey Mouse problem. Mickey Mouse lacks the necessary beneficence, ambivalence, and ambiguity, and we therefore lack the motivation and latitude to believe he is real.

## Supporting information

S1 Table The fitness relevance of distinct counterintuitive agents. The mean threat, benefit, and valence (absolute threat-benefit difference) of distinct religious and fictional agents, where N is the number of participants who named the agent in their list of five well-known beings and entities.
(DOC)
