# Supplementary material for: The Mickey Mouse problem: Distinguishing religious and fictional counterintuitive agents
Source: PLoS One. 2019 Aug 8;14(8):e0220886. doi: 10.1371/journal.pone.0220886 (PMC6687181; doi:10.1371/journal.pone.0220886)
Supplement: S1 Table — The mean threat, benefit, and valence (absolute threat-benefit difference) of distinct religious and fictional agents, where N is the number of participants who named the agent in their list of five well-known beings and entities. (DOC) [file pone.0220886.s001.doc]

***Religious Beings and Entities***

| **Agent** | **N** | **Threat** | **Benefit** | **Valence** |
| --- | --- | --- | --- | --- |
| God | 88 | 6.216 | 6.375 | 0.750 |
| Jesus | 88 | 5.125 | 6.148 | 1.386 |
| Zeus | 66 | 6.348 | 5.758 | 0.712 |
| Buddha | 64 | 3.250 | 5.109 | 1.953 |
| Allah | 33 | 5.667 | 5.697 | 1.061 |
| Satan | 30 | 6.400 | 2.933 | 3.467 |
| Muhammad | 25 | 3.800 | 4.200 | 1.440 |
| Ra | 15 | 6.867 | 6.267 | 0.600 |
| Odin | 13 | 6.000 | 5.923 | 0.846 |
| Shiva | 13 | 5.538 | 5.846 | 0.923 |
| Thor | 13 | 5.923 | 4.615 | 1.615 |
| Poseidon | 10 | 6.500 | 5.800 | 1.100 |
| Vishnu | 10 | 5.100 | 5.000 | 0.100 |
| Athena | 9 | 5.778 | 5.889 | 0.111 |
| Apollo | 8 | 5.500 | 6.500 | 1.000 |
| Krishna | 7 | 6.000 | 6.000 | 0.286 |
| Angel | 6 | 5.000 | 6.000 | 1.667 |
| Hades | 6 | 6.000 | 3.167 | 2.833 |
| Holy Spirit | 6 | 5.167 | 6.500 | 1.333 |
| Anubis | 5 | 6.600 | 6.400 | 1.000 |
| Aphrodite | 4 | 5.500 | 6.250 | 0.750 |
| Ares | 4 | 7.000 | 5.750 | 1.250 |
| Horus | 4 | 6.250 | 6.250 | 0.500 |
| Osiris | 4 | 6.250 | 6.250 | 0.000 |
| Demon | 3 | 7.000 | 2.000 | 5.000 |
| Ganesh | 3 | 6.667 | 6.667 | 0.000 |
| Hera | 3 | 7.000 | 6.667 | 0.333 |
| Jupiter | 3 | 7.000 | 5.667 | 1.333 |
| Loki | 3 | 7.000 | 5.333 | 1.667 |
| Zoroaster | 3 | 7.000 | 6.000 | 1.000 |
| Baal | 2 | 4.000 | 4.000 | 0.000 |
| Brahma | 2 | 5.000 | 6.500 | 1.500 |
| Hecate | 2 | 7.000 | 5.500 | 1.500 |
| Hercules | 2 | 7.000 | 7.000 | 0.000 |
| Hermes | 2 | 5.000 | 5.500 | 0.500 |
| Mother Nature | 2 | 4.000 | 4.000 | 0.000 |
| Rama | 2 | 6.500 | 6.500 | 0.000 |
| Venus | 2 | 5.500 | 7.000 | 1.500 |
| Ahriman | 1 | 7.000 | 2.000 | 5.000 |
| Amun | 1 | 7.000 | 6.000 | 1.000 |
| Antichrist | 1 | 7.000 | 1.000 | 6.000 |
| Artemis | 1 | 7.000 | 7.000 | 0.000 |
| Azura Mazda | 1 | 7.000 | 2.000 | 5.000 |
| Baphomet | 1 | 7.000 | 2.000 | 5.000 |
| Ceres | 1 | 7.000 | 7.000 | 0.000 |
| Djinn | 1 | 7.000 | 1.000 | 6.000 |
| Erebus | 1 | 7.000 | 4.000 | 3.000 |
| Gaia | 1 | 5.000 | 7.000 | 2.000 |
| Ghost | 1 | 4.000 | 1.000 | 3.000 |
| Hephaestus | 1 | 7.000 | 7.000 | 0.000 |
| Horace | 1 | 7.000 | 5.000 | 2.000 |
| Huitzilopochtli | 1 | 7.000 | 5.000 | 2.000 |
| Hydra | 1 | 7.000 | 1.000 | 6.000 |
| Indra | 1 | 6.000 | 6.000 | 0.000 |
| Isis | 1 | 1.000 | 4.000 | 3.000 |
| Kali | 1 | 7.000 | 4.000 | 3.000 |
| Kwan Yin | 1 | 7.000 | 7.000 | 0.000 |
| Mercury | 1 | 7.000 | 5.000 | 2.000 |
| Minerva | 1 | 1.000 | 1.000 | 0.000 |
| Neptune | 1 | 7.000 | 7.000 | 0.000 |
| Oshun | 1 | 1.000 | 2.000 | 1.000 |
| Pandora | 1 | 6.000 | 5.000 | 1.000 |
| Persephone | 1 | 5.000 | 1.000 | 4.000 |
| Prometheus | 1 | 7.000 | 7.000 | 0.000 |
| *Total/Mean* | *591* | *5.552* | *5.536* | *1.284* |

***Fictional Beings and Entities:***

| **Agent** | **N** | **Threat** | **Benefit** | **Valence** |
| --- | --- | --- | --- | --- |
| Superman | 88 | 6.295 | 6.159 | 0.909 |
| Spider-Man | 47 | 6.128 | 5.851 | 1.170 |
| Wonder Woman | 33 | 6.576 | 6.152 | 1.030 |
| Hulk | 30 | 6.800 | 4.967 | 1.900 |
| Wolverine | 19 | 6.684 | 5.368 | 1.421 |
| The Flash | 18 | 5.722 | 5.278 | 1.444 |
| Bigfoot | 16 | 6.563 | 1.625 | 4.938 |
| Santa Claus | 15 | 2.667 | 6.467 | 3.800 |
| Vampire | 15 | 6.733 | 3.133 | 3.600 |
| Werewolf | 13 | 6.538 | 2.077 | 4.462 |
| Dragon | 12 | 6.917 | 3.667 | 3.250 |
| Loch Ness Monster | 10 | 6.400 | 1.100 | 5.300 |
| Aquaman | 9 | 6.222 | 5.667 | 0.556 |
| Dracula | 9 | 6.889 | 3.333 | 3.556 |
| Fairy | 8 | 5.375 | 5.125 | 1.500 |
| Ghost | 8 | 5.250 | 3.875 | 1.625 |
| Unicorn | 8 | 4.375 | 3.875 | 2.000 |
| Green Lantern | 7 | 5.000 | 4.857 | 2.143 |
| Easter Bunny | 5 | 3.000 | 5.800 | 2.800 |
| Harry Potter | 5 | 6.600 | 6.400 | 0.600 |
| Leprechaun | 5 | 4.000 | 6.000 | 2.000 |
| Tooth Fairy | 5 | 4.000 | 5.000 | 1.000 |
| Dr. Strange | 4 | 7.000 | 6.500 | 0.500 |
| Magneto | 4 | 6.250 | 4.250 | 2.000 |
| Mermaid | 4 | 4.000 | 3.250 | 1.250 |
| Sasquatch | 4 | 7.000 | 1.500 | 5.500 |
| Zombie | 4 | 6.250 | 1.000 | 5.250 |
| Banshee | 3 | 6.000 | 1.333 | 4.667 |
| Darth Vader | 3 | 6.000 | 4.000 | 2.000 |
| Deadpool | 3 | 6.333 | 5.333 | 1.000 |
| Eleven | 3 | 6.667 | 4.000 | 2.667 |
| Genie | 3 | 4.667 | 7.000 | 2.333 |
| Godzilla | 3 | 7.000 | 1.000 | 6.000 |
| Mothman | 3 | 6.333 | 2.667 | 3.667 |
| Professor X | 3 | 7.000 | 6.333 | 0.667 |
| Chimera | 2 | 7.000 | 1.000 | 6.000 |
| Cyborg | 2 | 7.000 | 5.500 | 1.500 |
| Daredevil | 2 | 7.000 | 6.500 | 0.500 |
| Djinn | 2 | 7.000 | 4.000 | 3.000 |
| Dr. Manhattan | 2 | 7.000 | 7.000 | 0.000 |
| Elf | 2 | 3.500 | 4.500 | 2.000 |
| Gnome | 2 | 2.500 | 5.000 | 2.500 |
| Goblin | 2 | 7.000 | 1.500 | 5.500 |
| Jersey Devil | 2 | 7.000 | 1.000 | 6.000 |
| Legion | 2 | 5.500 | 5.000 | 0.500 |
| Leviathan | 2 | 7.000 | 4.000 | 3.000 |
| Rogue | 2 | 5.000 | 6.500 | 2.500 |
| Skinwalker | 2 | 7.000 | 1.000 | 6.000 |
| Storm | 2 | 5.000 | 6.000 | 3.000 |
| Superwoman | 2 | 4.000 | 4.000 | 1.000 |
| Terminator | 2 | 7.000 | 3.000 | 4.000 |
| Abarimon | 1 | 4.000 | 4.000 | 0.000 |
| Anthropos | 1 | 5.000 | 3.000 | 2.000 |
| Bloody Mary | 1 | 7.000 | 1.000 | 6.000 |
| Boogeyman | 1 | 7.000 | 1.000 | 6.000 |
| Captain Planet | 1 | 7.000 | 7.000 | 0.000 |
| Cerberus | 1 | 7.000 | 4.000 | 3.000 |
| Chupacabra | 1 | 6.000 | 2.000 | 4.000 |
| Constantine | 1 | 3.000 | 7.000 | 4.000 |
| Cthulhu | 1 | 7.000 | 5.000 | 2.000 |
| Cyclops | 1 | 7.000 | 4.000 | 3.000 |
| Dagon | 1 | 7.000 | 3.000 | 4.000 |
| Dr. Wells | 1 | 4.000 | 6.000 | 2.000 |
| Frankenstein | 1 | 7.000 | 3.000 | 4.000 |
| Ghost Rider | 1 | 7.000 | 4.000 | 3.000 |
| Ghoul | 1 | 7.000 | 3.000 | 4.000 |
| Goatman | 1 | 7.000 | 1.000 | 6.000 |
| Golem | 1 | 4.000 | 7.000 | 3.000 |
| Griffin | 1 | 7.000 | 1.000 | 6.000 |
| Hiro Nakamora | 1 | 7.000 | 7.000 | 0.000 |
| Hydro-Man | 1 | 3.000 | 3.000 | 0.000 |
| Ironfist | 1 | 7.000 | 5.000 | 2.000 |
| Luffy | 1 | 7.000 | 5.000 | 2.000 |
| Mangog | 1 | 3.000 | 3.000 | 0.000 |
| Mr. Freeze | 1 | 7.000 | 6.000 | 1.000 |
| Nazgul | 1 | 7.000 | 1.000 | 6.000 |
| Ninja Turtle | 1 | 3.000 | 4.000 | 1.000 |
| Nyarlathotep | 1 | 7.000 | 4.000 | 3.000 |
| Overmind | 1 | 5.000 | 5.000 | 0.000 |
| Peter Petrelli | 1 | 7.000 | 7.000 | 0.000 |
| Pixie | 1 | 5.000 | 7.000 | 2.000 |
| Predator | 1 | 7.000 | 4.000 | 3.000 |
| Q (Star Trek) | 1 | 6.000 | 7.000 | 1.000 |
| Red Flash | 1 | 7.000 | 7.000 | 0.000 |
| Sailor Moon | 1 | 4.000 | 3.000 | 1.000 |
| Sandman | 1 | 6.000 | 6.000 | 0.000 |
| Scarecrow | 1 | 7.000 | 4.000 | 3.000 |
| Seven of Nine | 1 | 7.000 | 4.000 | 3.000 |
| Shapeshifter | 1 | 7.000 | 1.000 | 6.000 |
| She-Ra | 1 | 5.000 | 5.000 | 0.000 |
| Silver Surfer | 1 | 7.000 | 7.000 | 0.000 |
| Skeletor | 1 | 6.000 | 4.000 | 2.000 |
| Slender Man | 1 | 7.000 | 2.000 | 5.000 |
| Sphinx | 1 | 6.000 | 4.000 | 2.000 |
| Starlord | 1 | 7.000 | 5.000 | 2.000 |
| Static Shock | 1 | 7.000 | 7.000 | 0.000 |
| Swamp Thing | 1 | 5.000 | 5.000 | 0.000 |
| Tarzan | 1 | 2.000 | 4.000 | 2.000 |
| The Invisible Man | 1 | 4.000 | 1.000 | 3.000 |
| The Mummy | 1 | 6.000 | 1.000 | 5.000 |
| The Stranger | 1 | 5.000 | 4.000 | 1.000 |
| The Thing | 1 | 7.000 | 3.000 | 4.000 |
| Weeping Angel | 1 | 7.000 | 2.000 | 5.000 |
| Wendigo | 1 | 7.000 | 1.000 | 6.000 |
| Witch | 1 | 5.000 | 6.000 | 1.000 |
| Wizard | 1 | 7.000 | 7.000 | 0.000 |
| Yeti | 1 | 7.000 | 1.000 | 6.000 |
| Yoda | 1 | 5.000 | 4.000 | 1.000 |
| *Total/Mean* | *518* | *6.054* | *4.780* | *2.135* |
